# Construction and Clarification of Dynamic Gene Regulatory Network of Cancer Cell Cycle via Microarray Data

**Published:** 2007-02-18

**Authors:** Cheng-Wei Li, Yung-Hsiang Chu, Bor-Sen Chen

**Affiliations:** Lab. of Systems biology, National Tsing Hua University, Hsinchu, 300, Taiwan

## Abstract

**Background:**

Cell cycle is an important clue to unravel the mechanism of cancer cells. Recently, expression profiles of cDNA microarray data of Cancer cell cycle are available for the information of dynamic interactions among Cancer cell cycle related genes. Therefore, it is more appealing to construct a dynamic model for gene regulatory network of Cancer cell cycle to gain more insight into the infrastructure of gene regulatory mechanism of cancer cell via microarray data.

**Results:**

Based on the gene regulatory dynamic model and microarray data, we construct the whole dynamic gene regulatory network of Cancer cell cycle. In this study, we trace back upstream regulatory genes of a target gene to infer the regulatory pathways of the gene network by maximum likelihood estimation method. Finally, based on the dynamic regulatory network, we analyze the regulatory abilities and sensitivities of regulatory genes to clarify their roles in the mechanism of Cancer cell cycle.

**Conclusions:**

Our study presents a systematically iterative approach to discern and characterize the transcriptional regulatory network in Hela cell cycle from the raw expression profiles. The transcription regulatory network in Hela cell cycle can also be confirmed by some experimental reviews. Based on our study and some literature reviews, we can predict and clarify the E2F target genes in G1/S phase, which are crucial for regulating cell cycle progression and tumorigenesis. From the results of the network construction and literature confirmation, we infer that MCM4, MCM5, CDC6, CDC25A, UNG and E2F2 are E2F target genes in Hela cell cycle.

## Introduction

The losses of cellular regulation give rise to most case of cancer. In cells, intricate genetic control systems regulate the balance between cell survival and death in response to growth signals, growth-inhibiting signals, and death signals. When some errors occur in the control systems causing cells to proliferate continuously, tumor just comes into being (Badawi et al. 2005).

The proliferation of cancer cell into two individual cells must go through cell cycle process (Whitfield et al. 2002; Valsesia-Wittmann et al. 2004). Cell cycle entails an ordered series of macromolecular events that lead to cell division and the production of two daughter cells. So that cell cycle is meaningful to the proliferation of cancer cell.

Expression levels of thousands of genes fluctuate throughout the cancer cell cycle (Cho et al. 2001; Ishida et al. 2001; Whitfield et al. 2002). Functional genes show periodic transcription to reflect cell growth, DNA synthesis, spindle pole body duplication and migration through the cell cycle (Cho et al. 1998). These processes and their regulation have been extensively investigated at the molecular level (Stillman, 1996; Nurse, 2000; Shah and Cleveland, 2000; Hinchcliffe and Sluder, 2001; Chen et al. 2004). Systems biology can be described as “integrative biology” with the ultimate goal of being able to predict de novo biological outcomes given the list of the components involved (Liu, 2005). Hence it is the coordinated study by (1) investigating the components of cellular networks and their interactions, (2) applying experimental high-throughput and whole-genome techniques, and (3) integrating computational methods with experimental effort (Klipp et al. 2005). In this situation, characterization of the genome-wide transcriptional program of the cell division cycle in mammalian cells is a critical step toward understanding the basic cell cycle processes and their roles in cancer. Therefore, it is worth investigating how these periodic patterns are regulated in the gene regulatory network of the cancer cell cycle from the systems biology perspective.

Gene expression data of Hela cell cycle have been collected (Whitfield et al. 2002) and analyzed with many clustering methods to organize which gene is associated with the cell cycle (Whitfield et al. 2002; Cho et al. 2001). Theoretically, it is possible to engineer the cell cycle network reversely, if we take cDNA expression levels as the output of gene expression networks, and collect cDNA expression levels of transcription factors as input. In order to realize how genes are regulated by transcription factors, we must also understand the interactions between target genes and their transcription factors (which transcription factor binds to which promoters). With all these information and interactive dynamic model, we get some clues to piece up the gene expression regulatory network in Hela cell cycle.

In this study, we attempt to devise an interactive dynamic model to characterize transcriptional regulatory network of the Hela cell cycle from the cDNA expression data of the human cell cycle in tumour (Whitfield et al. 2002). Based on our dynamic regulatory network, we not only predict the upstream regulators but also characterize the significance of the regulators depending on quantifying their regulatory abilities based on the corresponding biochemical kinetic parameters. At first, we construct a discrete-time dynamic model system and calculate the system kinetic parameters as the regulatory ability by using the expression data (Whitfield et al. 2002) and the system identification method (Johansson, 1993). Second, based on the interactive dynamic model, we detect the transcriptional regulatory function of target genes by the maximum likelihood parameter estimation method. Third, we trace back a group of upstream genes that play a role of transcriptional regulators of target genes in the Hela cell cycle of Homo sapiens via deducing the interactive relationship between the expression profiles of regulators and the detected transcription regulation of specific target genes. The pathway kinetic parameters of transcriptional regulatory network of Hela cell cycle are also estimated by the cDNA expression profiles of target genes and their upstream regulatory genes. Finally, these upstream regulatory genes are considered as the target genes. By a similar method, we construct their upstream regulatory genes one by one. Iteratively, we can construct the whole gene regulatory network of Hela cell cycle.

We applied our method to a publicly available data set of HeLa cell with microarray experiment on cell cycle (Whitfield et al. 2002) to identify the transcriptional regulators of cell cycle and to characterize their regulatory abilities on specific target genes. By means of the quantitative system analysis of the transcriptional regulatory network from Hela cell cycle genes, several transcription factors were identified and their regulatory abilities were determined. Further, some genes that may be suspected of regulators of Hela cell cycle are predicted here to be synergistic in harmonizing gene expression. Our proposed algorithm provides a novel approach to gain insight into the gene regulatory network of Hela cell by its gene expression data using system identification technique and discrete-time dynamic model. Furthermore, we combine the constructed Hela cell cycle dynamic network, some experimental reviews and E2F binding site’s research (Ren et al. 2002), we not only confirm the reliability of the dynamic network but also find the E2F target genes in Hela cell cycle progression. Finally, from the results of this study, we infer that E2F directly regulate MCM4, MCM5, CDC6, CDC25A, UNG and E2F2 in Hela cell cycle progression.

Our approach is so different from the statistical clustering method that it not only provides a suitable interactive dynamic model to decipher the complex signal transduction pathway that regulates gene expressions in Hela cell cycle but also predicts some potential regulators that have not been found. We can also quantify the regulatory abilities of the transcription factors by the corresponding kinetic activities to their target genes in the Hela cell cycle regulatory network. We could construct the cell cycle regulatory network in Hela cells quantitatively and discuss the sensitivity of regulatory genes to the gene regulatory network from the system analysis perspective.

## System Model and Network Identification

The construction of the transcriptional regulatory network of Hela cell cycle can be divided into two steps. First, the transcriptional regulation should be extracted from the gene expression data by the dynamic discrete-time model. Second, the upstream regulators will be traced back by correlating the transcriptional regulation of target genes with the expression profiles of possible regulators of Hela cell cycle. In this study, 64 transcription regulators, as shown in Table 1, are used as candidates of upstream regulators to each target gene. Finally, the kinetic parameters of gene transcriptional regulatory network of Hela cell are estimated by the cDNA expression profiles of target genes and their upstream regulatory genes.

### Dynamic signaling regulatory model

The second-order difference equation is used in the description of dynamic system evolved from the causality of gene regulatory function. Let *X_i_*(*k*) denote the expression profile of the *i*-th gene at time point *k*. The following second-order difference equation is proposed to model the cDNA expression level of the *i*-th gene,

(1) $$\begin{array}{c} X_{i}(k)+a_{i}X_{i}(k-1)+b_{i}X_{i}(k-2)\\ =G_{i}(k)+{ɛ}_{i}(k) \end{array}$$

where *G_i_*(*k*) is the upstream transcriptional regulatory function from regulatory genes to influence the expression profile *X_i_*(*k*) of the *i*-th gene while *a_i_* and *b_i_* are the parameters that characterize the dynamic inherent property of the gene like degradation and oscillation, and *ɛ_i_* (*k*) is the random noise of current microarray data or the residue of the model. In general, the second-order difference equation has been widely used to model dynamic discrete-time systems to efficiently characterize the dynamic properties of damping and resonance of systems in physics and engineering. The reason is that the roots of the characteristic equation of second-order dynamic equation may be a real double root, two real roots or conjugate roots, which could easily describe a system with undamping, overdamping, critical damping, under-damping or oscillation, dependent on the specification of their coefficients (Kreszig, 1993). These characteristics can not be easily described by the first-order dynamic equation. Therefore the second order stochastic equation is employed to characterize the biochemical processing of the gene expression.

Evidently, the transcriptional regulatory function *G_i_*(*k*) controls the synthetic rate of cDNA and the clue of upstream regulatory pathway is involved in *G_i_*(*k*). Therefore we emphasize on how to detect the upstream regulatory function *G_i_*(*k*) from expression data *X_i_*(*k*) and our dynamic model equation in equation (1). In general, it is not easy to extract transcriptional regulatory function *G_i_*(*k*). In order to extract the input regulatory function *G_i_*(*k*), we apply Fourier decomposition method to decompose *G_i_*(*k*) to generate some harmonic sinusoid functions. When the extraction problem is reduced to a simple parameter estimation problem, *G_i_*(*k*) can be decomposed by the following Fourier series.

(2) $$G_{i}(k)\approx \sum_{n=0}^{N}\left[\alpha_{n}\text{cos}(nk)+\beta_{n}\text{sin}(nk)\right]$$

Then we need to estimate the parameters of Fourier series, *α_n_* and *β_n_*, that are the magnitudes of different harmonics of sinusoid functions (cos(*nt*) and sin(*nt*)) for n = 0, …, N. Fourier series is a good tool to synthesize functions with finite energy by harmonic functions in respect of engineering.

### Extraction of the transcriptional regulatory function *G_i_*(*k*)

Since *G_i_*(*k*) has been decomposed, we combine equations (1) and (2) to get the following dynamic model equation for the expression profile of the *i*-th gene,

(3) $$\begin{array}{l} X_{i}(k)=-a_{i}X_{i}(k-1)-b_{i}X_{i}(k-2)\\ +\sum_{n=0}^{N}\left[\alpha_{n}\text{cos}(nk)+\beta_{n}\text{sin}(nk)]+{ɛ}_{i}(k\right) \end{array}$$

In the above dynamic model equation, the parameters *a_i_*, *b_i_*, *α_n_*, and *β_n_* should be estimated by the time profile of expression data of the *i*-th gene in linear scale, i.e. these parameters should be specified so that the simulating output *X*_i_(*k*) of the dynamic model in equation (3) should match the expression profile of the *i*-th gene. The maximum likelihood estimation method is employed to estimate these parameters *a_i_*, *b_i_*, *α_n_*, and *β_n_* in equation (3) from the expression profile *X_i_*(*k*) in the section Methods.

After the parameters *α_n_* and *β_n_* of the regulatory function *G_i_*(*k*) have been estimated in the section Methods, we can present the regulation detection *Ĝ_i_*(*k*)as follows,

(4) $${\hat{G}}_{i}(k)=\sum_{n=0}^{N}\left[{\hat{\alpha }}_{n}\text{cos}(nk)+{\hat{\beta }}_{n}\text{sin}(nk)\right]$$

where *α̂_n_* and *β̂_n_* are the estimates of *α_n_* and *β_n_*, respectively.

We know that the input transcriptional regulatory function *G_i_*(*k*) of the target gene of Hela cell cycle is often relative to the bindings of transcription factors or some interactions from the upstream regulators. In the next step, we will trace back the corresponding regulatory genes from the input regulatory function *Ĝ_i_*(*k*) of the target gene.

### Iterative algorithm for constructing gene regulatory network

In biology, the specific biochemical reactions are usually relative to the concentration of specific products. For this purpose, we describe the regulatory function as the following sigmoid function to describe the binding and nonbinding of transcription factors to motif binding sites (Chen et al. 2004; Klipp et al. 2005)

(5) $${\tilde{X}}_{j}(k)=\frac{1}{1+e^{-\gamma (X_{j}(k)-M_{j})}}$$

where γ is the transition rate and *M_j_* is the mean expression of the *j*-th regulatory gene’s profile *X_j_*(*k*).

We determine *R_i_* regulatory genes whose regulatory signals *X_j_*(*k*) *j* = 1, … , *R* are the most correlative to the target gene profile *X_i_*(*k*) of the *i-*th target gene. Then, we could reconstruct the gene regulatory network by tracing back the upstream regulators from the extracted regulatory function *Ĝ_i_* (*k*), which are contributed by *R_i_* regulatory genes, via the following biochemical kinetic relationship,

(6) $${\hat{G}}_{i}(k)=c_{i0}+\sum_{j\in R_{i}}c_{ij}{\tilde{X}}_{j}(k)+e_{i}(k)$$

where *c_ij_* is the pathway kinetic parameters from the regulatory gene *j* to target gene *i*, *R_i_* represents the number of the searched upstream regulatory genes selected by the absolute value of correlation coefficient between the target gene expression profile and the regulatory gene expression profile which is more than 0.8 based on the 95% confidence of normalized correlation coefficients of expression profiles of total cell cycle-related genes in Whitfield et al. 2002, the constant *c_i_*_0_ is the basal level denoting the regulatory function other than upstream regulatory genes, for example, due to post-transcriptional regulation, and *e_i_*(*k*) is the error or the noise of the network model.

Using the maximum likelihood algorithm in Method to estimate the parameters *c_i_*_0_ and *c_ij_* from *Ĝ_i_*(*k*) and *X_i_*(*k*), the regulation from the upstream regulators is identified as

(7) $${\hat{G}}_{i}(k)={\hat{c}}_{i0}+\sum_{j\in R_{i}}{\hat{c}}_{ij}{\tilde{X}}_{j}(k)$$

By combining equation (1) and the above equation, the dynamics of transcriptional regulatory network of the Hela cell cycle can be represented by the following identified difference equation,

(8) $$\begin{array}{c} X_{i}(k)=-{\hat{a}}_{i}X_{i}(k-1)-{\hat{b}}_{i}X_{i}(k-2)\\ +{\hat{c}}_{i0}+\sum_{j\in R_{i}}{\hat{c}}_{ij}{\tilde{X}}_{j}(k) \end{array}$$

where *i* = 1, 2, … for all profile target genes in Hela cell cycle.

In fact, equation (8) contains much information for exploring the regulatory network of each specific target gene of the Hela cell cycle. The regulatory genes, which belong to a specific set, *R_i_*, represent the potential upstream regulators for target gene *i*. The estimated chemical kinetic parameter, *ĉ_ij_*, characterizes the type and intensity of the influence of the *j*th regulatory gene on the *i*th target gene, in which positive sign indicates activation and a negative sign indicates repression, and the magnitude is defined as the regulatory ability. After the regulatory pathways of the *i*th target gene is constructed by tracing back their upstream regulatory genes, these *R_i_* upstream regulators are considered as target genes again to trace back their upstream regulatory genes. Iteratively, we can construct the whole gene regulatory network of the Hela cell cycle globally. The goal of reverse engineering gene regulatory network is to deduce the possible set of regulators and to identify their associated regulation abilities by the available data set from the dynamic system perspective. For this purpose, we devise a novel algorithm based on the dynamic gene expression model for searching possible upstream regulators and then identifying the relevant regulatory abilities *ĉ_ij_* according to equation (8).

## Results

### Data processing and analysis

Data were extracted by superimposing a grid over each array using GenePix 3.0 software (Axon Instruments). Spots of poor quality, determined by visual inspection, were removed from further analysis. Data of HeLa cell collected for each array were stored in the Stanford Microarray Database (SMD) and are available from SMD at http://genome-www5.stanford.edu/ (Sherlock et al. 2001; Whitfield et al. 2002).

We combine 775 cell cycle-related genes from the Human expression of Hela cell cycle-regulated genes according to the classification by Whitfield et al. (2002) with Human expression of cell cycle-regulated genes according to the traditional classification as the target genes. After that, we select 64 transcription factors (Table 1) from the 775 cell cycle genes (Whitfield et al. 2002).

**Figure 1:**
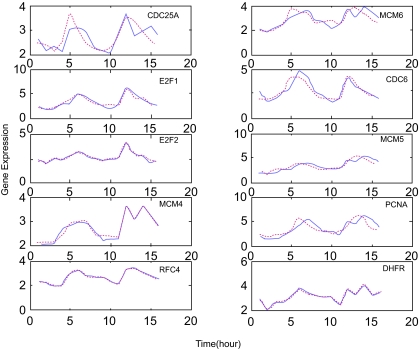
The second-order dynamic model fitting of E2F target genes The expression profile with the corresponding second-order dynamic model fitting for E2F target genes, CDC25A, MCM6, E2F1, CDC6, E2F2, MCM5, MCM4, PCNA, RFC4, and DHFR. The red dashed lines are the microarray data from Whitfield et al. 2002, and the blue solid lines are the estimated dynamic evolution of expression data by the proposed method. Obviously, the proposed second-order dynamic model could efficiently characterize the dynamic properties like damping and resonance of the gene regulatory network. The data have been transformed from the log scale in Whitfield et al. to linear scale.

**Figure 2:**
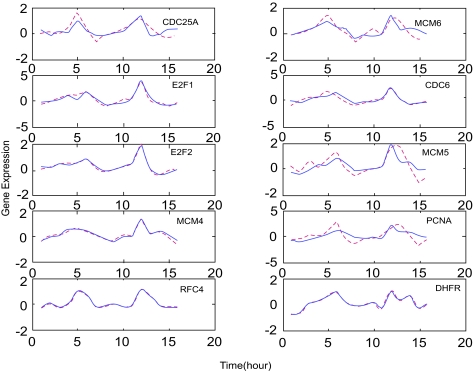
The extracted upstream regulatory functions from expression profiles (Whitfield et al. 2002) and their fitting by upstream regulatory genes The upstream transcriptional regulatory functions *Ĝ*(*t*) extracted from expression profiles of corresponding E2F target genes are denoted by the red dashed lines, and the blue solid lines are fitted by their upstream transcriptional regulatory functions in equation (6). These results show that the upstream TF regulatory model in equation (6) could match the regulatory function extracted from microarray data. In the figure, the gene expressions are expressed in linear scale.

The raw data were transformed into a linear scale from the original log ratio and applied to our approach. Following the dynamic model in equation (8), the parameters which characterize the dynamic regulatory mechanism are estimated successfully for each target gene in the pathway. Fig. 1 compares the simulation results of the dynamic expression model in equation (8) with the experimental expression profiles for some important cell cycle-related genes regulated by E2F family (Bracken et al. 2004), such as CDC25A (Stanelle et al. 2002; Muller and Helin, 2000; Ren et al. 2002), MCM6 (Ren et al. 2002; Polager et al. 2002; Ishida et al. 2001), E2F1 (Bracken, 2004), CDC6 (Stanelle et al. 2002; Ren et al. 2002), E2F2 (Ren et al. 2002; Muller et al. 2001), MCM5 (Ren et al. 2002; Ishida et al. 2001), MCM4 (Ren et al. 2002; Ishida et al. 2001), PCNA (Muller and Helin, 2000; Ren et al. 2002; Polager et al. 2002; Ishida et al. 2001), RFC4 (Ren et al. 2002; Polager et al. 2002), and DHFR (Ishida et al. 2001). The extracted regulatory functions, *Ĝ_i_*(*k*) of these genes which are estimated by the maximum likelihood algorithm in Methods from their expression profiles are shown in Fig. 2. The extracted regulatory function *Ĝ_i_*(*k*) in Fig. 2 are employed to estimate the kinetic parameters of gene regulatory network in equation (8) by the parameter estimation scheme in Methods. Our iterative algorithm can find the most likely regulatory genes that may participate in the expression program of Hela cell cycle genes.

## Inference of the regulatory pathway

**Figure 3:**
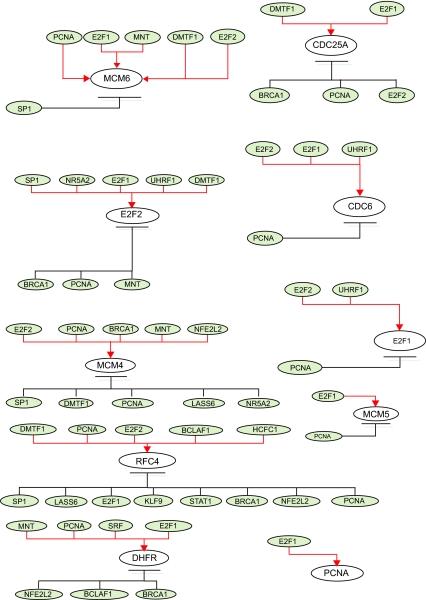
The regulatory pathways of E2F target genes in cancer cell cycle based on the dynamic regulatory modeling in Table 1 The related genes are represented as gray ovals and the regulatory abilities of the related regulators implying different degree of influence are converted into red-colored lines as positive regulations (activations) and black-colored lines as negative regulations (inhibitions) for each target gene. These regulatory pathways could be integrated as the regulatory network of cancer cell cycle in Figure 4.

For illustrations, the inferring strategy is applied to the E2F target genes (Bracken et al. 2004) in Hela cell cycle pathways to recognize their upstream regulatory genes. E2F transcription factors are well studied owing to their importance in both cell cycle (Muller and Helin, 2000; Nevins, 2001). Their regulatory abilities are shown in the upstream regulatory functions *Ĝ_i_*(*k*) of dynamic equation in Table 2. Parameters of regulatory functions *Ĝ_i_*(*k*) in Table 2 represent the regulatory abilities and sensitivities of the relative transcription factors. It is very exciting that E2F1 and E2F2 are found to be active regulators in most E2F target genes listed in Table 2, which agree very well with the previous results (Cam and Dynlacht, 2003; Ivey-Hoyle et al. 1993; Lang et al. 2001). The regulatory abilities of the related regulators implying different degrees of influence are converted to the red-colored lines as positive regulations (activations) and the black-colored lines as negative regulations (inhibitions) for each target gene. Then, based on the dynamic regulatory equations in Table 2 (see detail in Supplementary Table S1), the pathways of E2F target gene in Hela cell cycle regulatory system are described in Fig. 3. The coefficients of these dynamic regulatory equations represent the kinetic activities of regulatory genes. If a regulatory gene is with a large kinetic parameter in the dynamic regulatory equation, it will play an important role in Hela cell cycle and is more sensitive to the gene expression of target gene.

Based on the dynamic regulatory modeling, the 152 E2F target genes are found and shown in Table 3. In these 152 E2F target genes, 6 genes match the E2F target genes found by Elkon et al. (2003) from 124 E2F target genes edited by Ren et al. (2002) and 17 genes match the E2F target genes found by Elkon et al. (2003) from 872 periodic genes edited by Whitfield et al. (2002). Ren et al. (2002) has found 124 E2F target genes with E2F binding promoter. The comparisons of these results are shown in Fig. 9.

**Figure 4:**
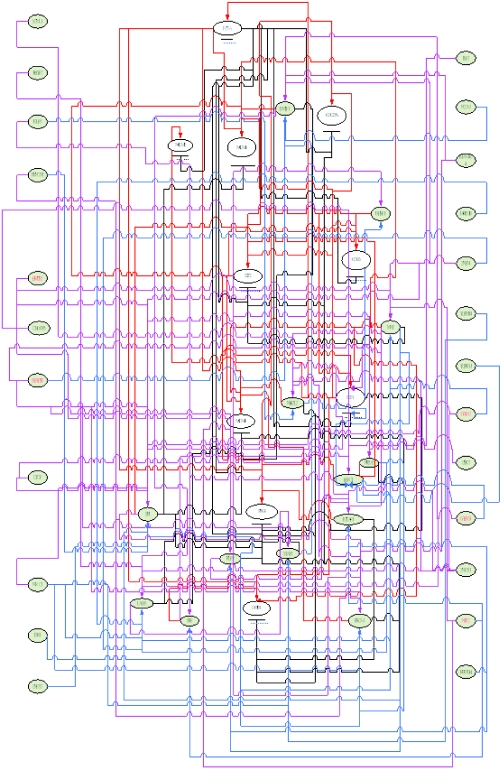
The network of E2F target genes in cancer cell cycle based on the dynamic regulatory modeling The regulatory pathway of the *i* th target gene is constructed by tracing back its upstream regulatory genes. Then, these upstream regulatory genes are considered as target genes again to trace back their upstream regulatory upstream regulatory genes. Iteratively, we can construct the whole gene regulatory network of cancer cell cycle globally. In the first layer of the upstream regulators, red solid lines represent positive regulation and black solid lines represent negative regulation, and in the second layer of the upstream regulators, pink solid lines represent positive regulation and blue solid lines represent negative regulation. Based on this procedure, this E2F regulatory network is the interaction of regulatory pathways in Fig. 3. **The enlarged version of **Figure 4 and the more clear figures of this paper are provided in http://www.ee.nthu.edu.tw/bschen/Li.htm.

After finishing the construction of the first layer network of E2F target genes (Bracken et al. 2004), we take these upstream regulators as target genes. By using similar method, we construct the upstream regulatory genes of these target genes. In the second layer network, the regulatory abilities implying different degrees of influence are converted into pink-colored lines as positive regulations (activations) and blue-colored lines as negative regulations (inhibitions). Then, we combine the first layer network and the second layer network together to form a more complete network to E2F target genes in HeLa cell cycle as described in Fig. 4. Iteratively, we can construct the higher layer network to complete the gene regulatory network of Hela cell cycle.

## Discussion

The losses of cellular regulation give rise to most cases of cancer. The transcription factors are crucial for regulating cell cycle progression and may be related to the development of a cancer. Therefore, to understand these gene regulatory processes, we need to unravel the regulatory mechanisms of these transcription factors in cell cycle. Our study presents a systematically iterative approach to discern and characterize the transcriptional regulatory network of 775 cell cycle-related genes from the raw expression profiles of Hela cell (Whitfield et al. 2002). Because the transcriptional regulatory network of 775 cell cycle-related genes is very complicated, two miniature gene regulatory networks of E2F family during G1/S phase in Fig. 4 and the other family during G2/M in Fig. 8 are given to illustrate the regulatory mechanism of Hela cell.

**Figure 5:**
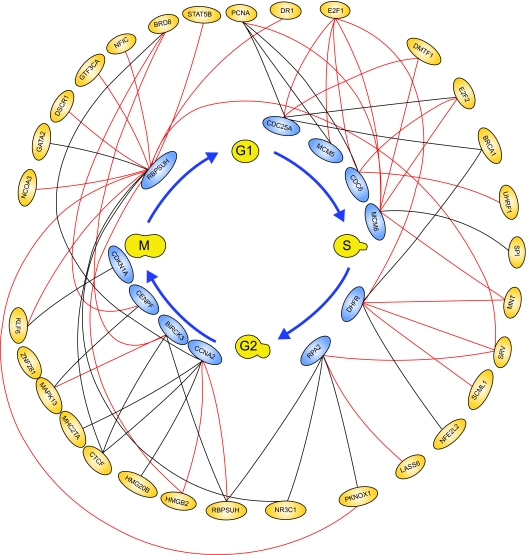
The miniature cancer cell cycle network In this figure we just list some E2F target genes and show the role of E2F transcription factors in cancer cell cycle (Muller and Helin 2000). Red solid lines represent positive regulations and black solid lines represent negative regulations.

Our approach also offers the following advantages. First, based on the dynamic regulatory model, a gene regulatory network of cancer cell could be constructed by the extracted upstream regulatory function through microarray data. Then, the identified regulatory ability for each specific regulator could evaluate the contribution of this regulator; the positive sign stands for activation and the negative sign stands for repression, and the magnitude represents the significance. These advantages of the proposed approach will improve the analysis to cope with rapidly growing microarray data of human. BRCA1 is one of the important cell cycle-related genes to play as a transcriptional repressor in cell cycle progress (Kennedy et al. 2005). This finding matches the gene regulatory network constructed by dynamic regulatory model (shown in Fig. 4 and Fig. 5). It is clear that E2F regulates the expression of a host of factors that function during G1/S transition and S phase even the whole cell cycle (Bracken et al. 2004). E2F is best known for its role in regulating the transcription of genes that positively affect cell cycle progression (Ortega et al. 2002; Sherr and Roberts, 1999; Di et al. 2003; Stott et al. 1998; Trimarchi and Lees, 2002; Hayashi et al. 2006).

Our results almost match this finding (shown in Fig. 4 and Fig. 5). Hayashi with his collaborator found that E2F1 activates human MCM8. In our study, we found that E2F1 activates human MCM5 and MCM6 (shown in Fig. 4 and Fig. 5). Elkon et al. (2003) and Ren et al. (2002) have found that human MCM5 and MCM6 are two E2F target genes (shown in Table 3). As a result of these studies, we infer that human MCM5 and MCM6 may be positively regulated directly by E2F1 in Hela cell cycle. Accumulating evidence indicates that cdc25A possesses oncogenic properties. Recently, overexpression of cdc25A was found in many breast, head and neck cancers (Wu et al. 1998). CDC6 plays a critical role in the regulation of the onset of DNA replication in eukaryotic cells and Cdc6 expression is down-regulated in prostate cancer (Robles et al. 2002). From the results of Table 3, we could infer that E2F directly regulates MCM4, MCM5, CDC6, CDC25A, UNG and E2F2 in Hela cell cycle progression. In Fig. 5, we represent some E2F target genes and show the importance of E2F transcription factor in Hela cell cycle progression. In Fig. 7, we also predict the probable transcription regulations in some target genes, which express in G2/M phase of human cell cycle as shown in the regulatory network of Fig. 8. Further, we can construct not only E2F related regulatory network but also the whole Hela cell cycle network if we have genome-wide microarray data and CHIP data. However, at present, we still need to get enough evidence and CHIP experiments in the same cellular system to confirm our gene regulatory network of Hela cell cycle (Bracken et al. 2004).

Based on the dynamic regulatory modeling, the 152 E2F target genes are found and shown in Table 3. These target genes may be regulated by E2F directly or indirectly. In these 152 E2F target genes, 6 genes match the E2F target genes found by Elkon et al. (2003) from 124 E2F target genes with E2F promoter edited by Ren et al. (2002) and 17 genes match the E2F target genes found by Elkon et al. (2003) from 872 periodic genes in Hela cell cycle edited by Whitfield et al. (2002).

**Figure 6:**
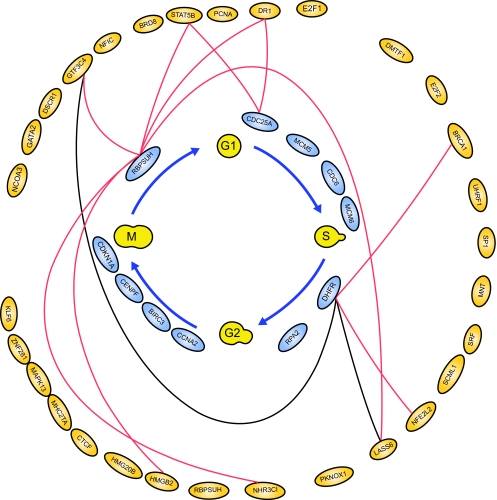
The miniature cancer cell cycle network in Fig. 5 is repeated as independent validation by randomly reshuffling the time order of microarray experiment but with the same choices of target and regulatory genes Obviously, the proposed regulatory network in Fig. 5 is destroyed by the reshuffling of experimental data. Red solid lines represent positive regulations and black solid lines represent negative regulations. For example, BRCA1 in Fig. 5 plays as a transcriptional repressor in cell cycle progress (Kennedy et al. 2005). From Fig. 6, BRCA1 becomes a transcriptional activator.

**Figure 7:**
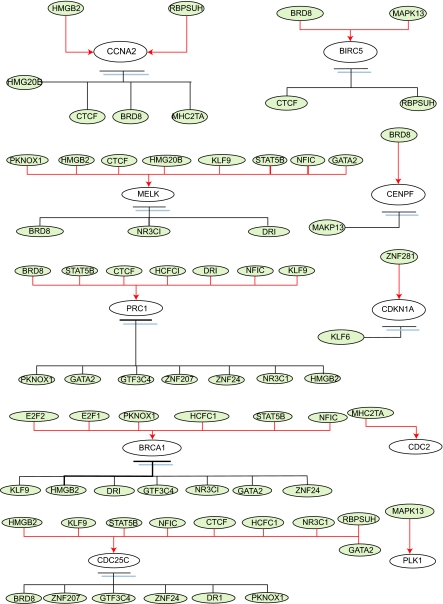
The regulatory pathways of target genes, which expressed in G2/M phase of human cell cycle, are based on the dynamic regulatory modeling The related genes are represented as gray ovals and the regulatory abilities of the related regulators implying different degree of influence are converted into red-colored lines as positive regulations (activations) and black-colored lines as negative regulations (inhibitions) for each target gene.

**Figure 8:**
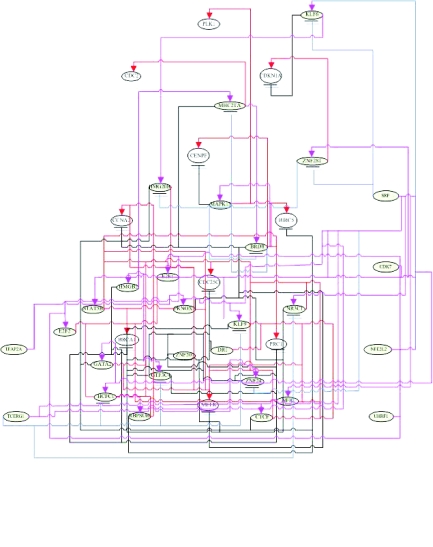
The gene regulatory network of in G2/M phase of cancer cell cycle based on the dynamic regulatory modeling and the interactions of regulatory pathways in Fig. 7.

Finally, in order to validate the proposed approach, an independent validation is also given by randomly reshuffling the time order of micro-array experiment but with the same choices of target gene and regulatory genes, to confirm the reliability of the proposed method as shown in Fig. 6. As previous statement, BRCA1 plays as a transcriptional repressor in cell cycle progress (Kennedy et al. 2005) (shown in Fig. 5). From the shuffling results shown in Fig. 6, BRCA1 becomes a transcriptional activator. It is clearly seen that the proposed Hela cell cycle regulatory pathway in Fig. 5 is destroyed by reshuffling the experimental data.

## Methods

### Maximum likelihood Estimation of *a_i_*, *b_i_*, *α_n_* and *β_n_*

The dynamic equation (3) must match the expression profile at all time points and then is arranged in a vector difference form. Consequently, the vector dynamic form of this equation is applied to m time points of expression profile in order to make the dynamic model work.

(M.1) $$\begin{array}{l} X_{ik}=-a_{i}X_{ik-1}-b_{i}X_{ik-2}\\ +\sum_{n=0}^{N}[\alpha_{n}\text{cos}(n)+\beta_{n}\text{sin}(n)]+{ɛ}_{ik} \end{array}$$

$$\begin{array}{l} X_{im}=\left[\begin{array}{c} X_{i}(3)\\ X_{i}(4)\\ \vdots \\ X_{i}(m) \end{array}\right],\;X_{im-1}=\left[\begin{array}{c} X_{i}(2)\\ X_{i}(3)\\ \vdots \\ X_{i}(m-1) \end{array}\right],\\ X_{im-2}=\left[\begin{array}{c} X_{i}(1)\\ X_{i}(2)\\ \vdots \\ X_{i}(m-2) \end{array}\right], \end{array}$$

where

$$\begin{array}{l} \text{cos}(n)=\left[\begin{array}{c} \text{cos}(n3)\\ \text{cos}(n4)\\ \vdots \\ \text{cos}(nm) \end{array}\right],\;\text{sin}(n)=\left[\begin{array}{c} \text{sin}(n3)\\ \text{sin}(n4)\\ \vdots \\ \text{sin}(nm) \end{array}\right],\\ {ɛ}_{im}=\left[\begin{array}{c} {ɛ}_{i}(3)\\ {ɛ}_{i}(4)\\ \vdots \\ {ɛ}_{i}(m) \end{array}\right]. \end{array}$$

Next, we translate equation (M.1) into a matrix form,

(M.2) $$Y_{i}=A_{i}\Phi_{i}+E_{i}$$

where Φ*_i_* = [*a_i_* *b_i_* *α*_0_ *β*_0_ ··· *α_N_* *β_N_*]*^T^* and *E_i_* = *ɛ_im_* are in vector forms, and *A_i_* = [−*X_im_*_−1_ − *X* *_im_*_−2_ cos(0) sin(0) ··· cos (N) sin (N)]^T^ is a matrix.

Then we use the maximum likelihood estimation to derive the optimal parameters estimation of Φ̂*_i_* (Johansson, 1993).

We assume that each element in the error vectors, *ɛ_i_*(*k*),*k* = {3, ... , *m*}, is an independent random variable with a normal distribution with zero mean and variance *σ*^2^, and we will estimate the parameter Φ̂*_i_* , by the maximum likelihood method.

(M.3) $$\begin{array}{l} p(Y_{i}|\Phi_{i},\sigma^{2})=\frac{1}{\sqrt{2\pi \sigma^{2}}}\text{exp}\\ \left\{-\frac{{\left[Y_{i}-A_{i}\Phi_{i}\right]}^{T}[Y_{i}-A_{i}\Phi_{i}]}{2\sigma^{2}}\right\} \end{array}$$

The log-likelihood function for given *m* data points is then described by–

(M.4) $$\begin{array}{l} \text{log}L(\Phi_{i},\sigma^{2})=-\frac{m-2}{2}\text{ln}[2\pi \sigma^{2}]\\ -\frac{1}{2\sigma^{2}}\sum_{k=1}^{m}{\left[Y_{i}-A_{i}\Phi_{i}\right]}^{T}[Y_{i}-A_{i}\Phi_{i}] \end{array}$$

Here we expect the log-likelihood function to have the maximum at Φ = Φ̂ and *σ*^2^ = *σ̂ *^2^. The necessary condition for maximum likelihood estimates Φ̂ and *σ̂*^2^ as follows (Johansson, 1993).

(M.5) $$\begin{array}{l} {\frac{\partial \text{log}L(\Phi ,\sigma^{2})}{\partial \Phi }|}_{\Phi =\hat{\Phi }}=0\\ {\frac{\partial \text{log}L(\Phi ,\sigma^{2})}{\partial \sigma^{2}}|}_{\sigma^{2}={\hat{\sigma }}^{2}}=0 \end{array}$$

The estimated parameters Φ̂ and *σ̂*^2^ are shown below.

(M.6) $${\hat{\Phi }}_{i}={\left(A_{i}^{T}A_{i}\right)}^{-1}A_{i}^{T}Y_{i}$$

(M.7) $${\hat{\sigma }}^{2}=\frac{1}{m-2}{\left(Y_{i}-A_{i}\Phi_{i}\right)}^{T}(Y_{i}-A_{i}\Phi_{i})$$

Theoretically, *E_i_* is just the noise of the gene expression profile of the microarray chips, but some modeling errors and approximation errors in equation (2) are also involved in *E_i_*. So that taking the modeling error and approximation error in our consideration makes our dynamic model equation more approach the actual situation. The number of Fourier series *N* is determined by tradeoff between the computational complexity of parameter estimation in equation (M.6) and the accuracy of approximation in equation (2). According to our expression data, we choose *N* = 16 to make the synthesis of these harmonics be the best approximation to the expression data.

## Parameter Estimation of *c_i_*_0_ and *c_ij_*

To estimate the pathway kinetic parameters *c_ij_* in equation (6) by *Ĝ_i_*(*k*) and *X̃_i_*(*k*) with *m* time points of upstream regulatory expression profile, equation (6) is represented with algebraic form as follows,

(M.8) $${\hat{G}}_{i}=B_{i}\Omega_{i}+V_{i}$$

$${\hat{G}}_{i}=\left[\begin{array}{c} {\hat{G}}_{i}(3)\\ {\hat{G}}_{i}(4)\\ \vdots \\ {\hat{G}}_{i}(m) \end{array}\right]\;B_{i}=\left[\begin{array}{ccccc} 1 & {\tilde{X}}_{1}(3) & \cdots & \cdots & {\tilde{X}}_{R_{i}}(3)\\ \vdots & \vdots & \ddots & & \vdots \\ \vdots & \vdots & & \ddots & \vdots \\ 1 & {\tilde{X}}_{1}(m) & \cdots & \cdots & {\tilde{X}}_{R_{i}}(m) \end{array}\right],$$

where

$$\Omega_{i}=\left[\begin{array}{c} c_{i0}\\ c_{i1}\\ \vdots \\ c_{iR_{i}} \end{array}\right],\;\text{and }V_{i}=\left[\begin{array}{c} e_{i}(3)\\ e_{i}(4)\\ \vdots \\ e_{i}(m) \end{array}\right]$$

We assume that each element in the error vectors, *e_i_*(k), *k* = {3,..., *m*}, is an independent random variable with a normal distribution with zero mean and variance *σe_i_*^2^. Then by the similar procedure, the estimated parameters Ω̂ and*σe_i_*^2^ are shown below.

(M.9) $$\hat{\Omega }={\left(B_{i}^{T}B_{i}\right)}^{-1}B_{i}^{T}{\hat{G}}_{i}$$

(M.10) $$\sigma_{e_{i}}^{2}=\frac{1}{m-2}{\left({\hat{G}}_{i}-B_{i}\Omega_{i}\right)}^{T}({\hat{G}}_{i}-B_{i}\Omega_{i})$$

## Supplementary Material

Supplementary Table S1 A miniature dynamic model network with the identified upstream regultors and their downstream target genes in the pathway of E2F target genes in cancer cell cycle. The coefficients characterize the corresponding regulatory abilities and sensitivities of the transcription regulations. The positive sign implies activations while the negative sign implies inhibitions for each target gene.

## Figures and Tables

**Table 1:** CloneIDs (Whitfield et al. 2002) of 64 transcription factors in human cell cycle are selected as candidates to regulate downstream target genes Gene names are boldly denoted behind or under the CloneIDs in brackets. IEA denotes Inferred from Electronic Annotation in NCBI.

IMAGE:173309**(BCLAF1)**	IMAGE:564803**(FOXM1)** IEA	IMAGE:590774**(MAPK13)** Knebel et al.	IMAGE:202704**(SCML1)** IEA
IMAGE:241474**(BRCA1)** IEA	IMAGE:149809**(GATA2)** IEA	IMAGE:1536451 **(MHC2TA)**	**IMAGE**:782622 **(SP1)**
IMAGE:815287**(BRD8)** Monden et al.	IMAGE:135688**(GATA2)** IEA	IMAGE:809731**(MNT)** Meroni et al.	IMAGE:80318 **(SP1)**
IMAGE:825210**(C14orf106)** IEA	IMAGE:780958 **(GTF3C4)**	IMAGE:197520**(NCOA3)** IEA	IMAGE:840636 **(SRF)**
IMAGE:726588**(C14orf106)** IEA	IMAGE:291827 **(GTF3C4)**	IMAGE:884438**(NFE2L2)** IEA	IMAGE:545503 **(STAT1)**
IMAGE:1915416**(CDK7)** Shiekhattar et al.	IMAGE:344049**(HCFC1)** Wysocka et al.	IMAGE:1455463 **(NFIC)**	IMAGE:134120 **(STAT5B)**
IMAGE:130242**(CDK7)** Shiekhattar et al.	IMAGE:897806 **(HIF1A)**	IMAGE:265874 **(NFIC)**	IMAGE:712840 **(STAT5B)**
IMAGE:268652**(CDKN1A)** IEA	IMAGE:878184**(HMG20B)** IEA	IMAGE:271198**(NR3C1)** Takahashi et al.	IMAGE:132857 **(STAT5B)**
IMAGE:240367**(CTCF)** Filippova et al.	IMAGE:1842250**(HMGB2)** Shirakawa et al.	IMAGE:245517**(NR5A2)** IEA	IMAGE:272192 **(TCERG1)**
IMAGE:490728 **(DMTF1)**	IMAGE:363103**(HMGB2)** Shirakawa et al.	IMAGE:43229**(PCNA)** IEA	IMAGE:137387 **(TFAP2A)**
IMAGE:487797 **(DR1)**	IMAGE:242952**(ILF2)** Reichman et al.	IMAGE:789182**(PCNA)** IEA	IMAGE:868630**(TGFB1I4)** IEA
IMAGE:566760 **(DR1)**	IMAGE:838829**(JARID1B)** IEA	IMAGE:30114 **(PHTF2)**	IMAGE:366414**(UHRF1)** IEA
IMAGE:884462**(DSCR1)** Fuentes et al.	IMAGE:345056**(KIAA1404)** IEA	IMAGE:1947972**(PKNOX1)** Chen et al.	IMAGE:1550739**(UHRF1)** IEA
IMAGE:236142 **(E2F1)**	IMAGE:510381**(KLF6)** Ratziu et al.	IMAGE:2018976**(PTTG1)** Dominguez et al.	IMAGE:246869**(ZNF207)** Pahl et al.
IMAGE:768260 **(E2F1)**	IMAGE:302549**(KLF9)** Imataka et al.	IMAGE:781089**(PTTG1)** Dominguez et al.	IMAGE:296429**(ZNF24)** IEA
IMAGE:293331 **(E2F2)**	IMAGE:35147**(LASS6)** Banerjee-Basu et al.	IMAGE:845502**(RBPSUH)** Hsieh et al.	IMAGE:280750**(ZNF281)** Law et al.

**Table 2:**
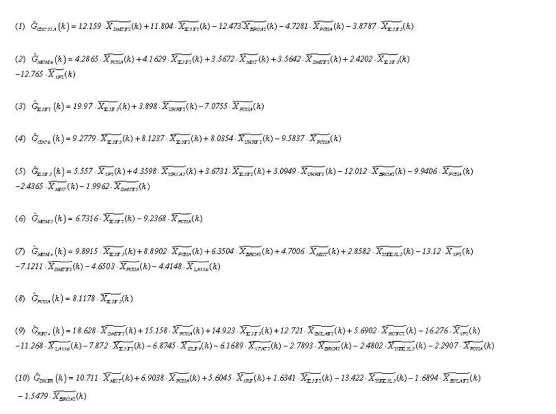
Upstream regulatory TFs and their regulatory function *Ĝ*(*k*) on E2F target genes in cancer cell cycle The positive sign implies activations while the negative sign implies inhibitions for each target gene. The magnitudes indicate their regulatory abilities to the downstream target genes.

**Table 3:** The 152 E2F target genes in the gene regulatory network based on the dynamic regulatory modeling The marked signs are the matched E2F target genes found by Elkon et al. (2003).

BAIAP2	TOP3A	ACYP1	BIVM	ADCK2	OGT	C20orf55
SSR3	MED31	CDC6*#	FLJ10154	FANCG	PILRB	MET
SLBP#	NASP	ADCY6	MGC15716	APEX2	FLJ11021	NR5A2
MNT	CDC25A*#	ANKRD10	ABCA7	LOC197336	ASF1B	CDKN2D
LOC221955	UNG*#	BARD1	FLJ13912	RBBP8	SFRS5#	CENPE
LOC90110	MGC3207	E2F2*	Pfs2	MCM8	C22orf18	USP16
FBXL20	UBQLN2	MCM5*#	EGFL5	C20orf111	LOC58486	ARHGAP8
ANKRD25	RAMP	CCRK	NKTR	ABCC5	MAP3K2	PSEN1
KIAA1529	MCM6*#	TREX1	KIAA0092	MLF1IP	EZH2	C6
PTOP	DMTF1#	RAB23#	LOC200895	FANCA	CENTB5	USP6NL
TRIM26	KIAA0738	FLJ10618	HSPB8	DNAJC6	HIST3H2A	LOC51334
ZMYND19	PNN	OACT1	C14orf130	RAD18	BRCA1	AKAP13
CDCA7	MAP2K6	ORC1L#	KIAA1586	SP1	FLJ35740	AGPAT3
CASP8AP2#	GMNN	MCM4#	HELLS	USP1	HSPC150	ASAM
PLCXD1	LOC283596	MSH2	CLSPN	MYCBP2	ORC3L	RAB3A
ZNF367	CASP2	LCHN	RFC4	TOPBP1#	DNAJB4	HIST2H2BE
RNP	C9orf42	FLJ20280	DONSON	DHFR	RRM1	HIST2H2AA
USP53	E2F1#	PCNA	AP4B1	DKFZP434B168#	RHOBTB3	H2AFY
SKP2	CCNE2#	MBD4	MGC2610	DLNB14	KIAA0841	H3F3A
PDXP	SCML1	TLOC1	ATAD2	RFC2	NFE2L2	HIST1H3D
FLJ20530	SERPINB3	SDC1	RPA2#	NSUN3	BBS2	
PANK2	UHRF1	PRPS2	FEN1	FKBP6	FANCD2	

* E2F target genes found by Elkon et al. (2003) from 124 E2F target genes edited by Ren et al. (2002).

# E2F target genes found by Elkon et al. (2003) from 872 periodic genes edited by Whitfield et al. (2002).
